# Assessment of the influence of levees along Yangtze River on *Oncomelania hupensis*, the intermediate host of *Schistosoma japonicum*

**DOI:** 10.1186/s13071-024-06318-1

**Published:** 2024-07-07

**Authors:** Shen Chen, Yin-Long Li, Lei Duan, Jian-Bing Liu, Jie Zhou, Dan-Dan Lin, Shi-Qing Zhang, Kun Yang, Li-Yong Wen, Yan-Jun Jin, Shang Xia, Jing Xu, Shan Lv, Shi-Zhu Li, Xiao-Nong Zhou

**Affiliations:** 1 National Institute of Parasitic Diseases, China CDC (Chinese Center for Tropical Diseases Research) Key Laboratory On Parasite and Vector Biology, National Health Commission WHO Collaborating Centre for Tropical Diseases, National Center for International Research On Tropical Diseases, Ministry of Science and Technology, Shanghai, 200025 China; 2 Fudan University School of Life Sciences, Shanghai, 200438 China; 3 Hubei Provincial Center for Disease Control and Prevention, Wuhan, 430079 China; 4 Hunan Institute for Schistosomiasis Control, Yueyang, 414021 China; 5 Jiangxi Provincial Institute of Parasitic Diseases, Nanchang, 330046 China; 6 Anhui Provincial Institute of Parasitic Diseases, Hefei, 230061 China; 7 Jiangsu Provincial Institute of Parasitic Diseases, Wuxi, 214064 China; 8 Zhejiang Provincial Center for Schistosomiasis Control, Hangzhou Medical College, Hangzhou, 310007 China; 9 Shanghai Municipal Center for Disease Control and Prevention, Shanghai, 200336 China; 10 School of Global Health, Chinese Center for Tropical Diseases Research, Shanghai Jiao Tong University School of Medicine, Shanghai, 200025 China

**Keywords:** *Oncomelania hupensis*, Schistosomiasis, Levee, Flooding, Yangtze River, China

## Abstract

**Background:**

*Oncomelania hupensis* is the exclusive intermediate host of *Schistosoma japonicum* in China. Snail control is an essential component of schistosomiasis elimination programme. With 70 years of continuous efforts, the range of *O. hupensis* had reduced significantly, but slowed down in last decades. A large number of levees against flooding were constructed along Yangtze River and its affiliated lakes in the middle and lower reaches, which influenced the hydrology and ecology in the alluvial plains. The purpose of this study was to assess the impact of levees on the distribution of *O. hupensis* in the middle and lower reaches of the Yangtze River.

**Methods:**

The snail habitats were digitalised by hand-held GPS system. The years for discovery and elimination of snail habitats were extracted from historical records. The accumulated snail-infested range for each habitat was calculated on the basis of annual reports. The current distribution of *O. hupensis* was determined by systematic and environmental sampling. The geographical distribution of levees was obtained from satellite imagery. To assess the impact of levees, the data pertaining to *O. hupensis* were divided into two parts: inside and outside the Yangtze River. Joinpoint regression was utilised to divide the study time span and further characterise the regression in each period. The 5-year-period moving averages of eliminated area infested by snails were calculated for the habitats inside and outside Yangtze River. The moving routes of corresponding geographical median centres were simulated in ArcGIS. Hotspot analysis was used to determine the areas with statistical significance clustering of *O. hupensis* density.

**Results:**

Three periods were identified according to Joinpoint regression both inside and outside Yangtze River. The area infested by *O. hupensis* increased in the first two periods. It decreased rapidly outside Yangtze River year over year after 1970, while that inside the Yangtze River did not change significantly. Furthermore, the latter was significantly higher than the former. It was observed that the present density of *O. hupensis* inside Yangtze River was lower than outside the Yangtze River. The median centre for eliminated ranges inside Yangtze River wavered between the east (lower reach) and the west (middle reach). In contrast, the median centre for eliminated ranges continuously moved from the east to the west.

**Conclusions:**

Our findings indicated that the levees had a considerable negative impact on the distribution of *O. hupensis* outside Yangtze River. Some hotspots observed in the irrigation areas need a sluice system at the inlet of branch for snail control. The major distribution of *O. hupensis* located in Hubei might be caused by severe waterlogging. The intensive surveillance should be implemented there. The biggest two freshwater lakes, the major endemic regions historically, were identified as cold spots. The long-term impact of Three Gorges Dam on the distribution of *O. hupensis* in the lakes should be monitored and evaluated.

**Graphical Abstract:**

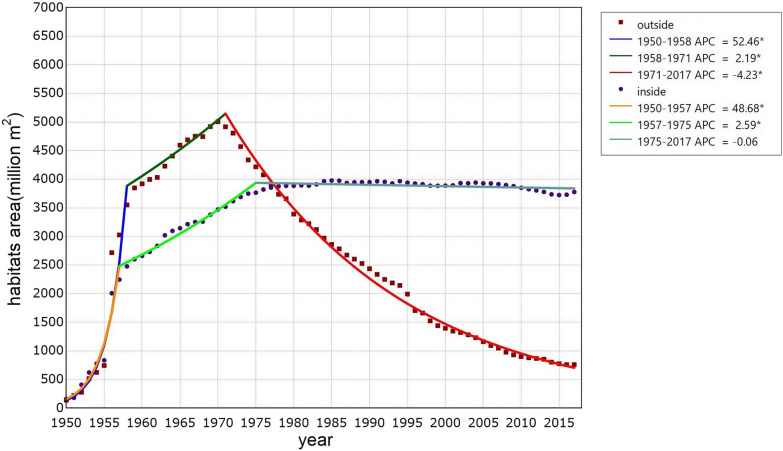

**Supplementary Information:**

The online version contains supplementary material available at 10.1186/s13071-024-06318-1.

## Background

Human schistosomiasis is endemic in 78 countries and 251.4 million people in 51 countries require preventive chemotherapy [1]. Schistosomiasis is a targeted disease that will be eliminated in the WHO road map for neglected tropical diseases 2021–2030 [2]. All endemic countries are expected to eliminate schistosomiasis as a public health problem by 2030. Furthermore, 25 countries will eliminate the disease. Asian schistosomiasis is mainly caused by *Schitosoma japonicum* and *S. mekongi* [3]. The former is endemic in the People’s Republic of China (P.R. China), the Philippines, Indonesia and Japan. The latter prevails in Cambodia and Laos. Japan achieved transmission interruption in 1974 [4], and five other countries are in the list of countries which will achieve transmission interruption of schistosomiasis by 2030.

P.R. China is one of the countries most affected by schistosomiasis. It was estimated that 9.49 million human cases and 120,000 sick cattle occurred in the late 1950s [5]. The national programme of schistosomiasis control, launched in the mid-1950s, has made a difference. At present, about 30,000 advanced cases exist in the country, but only few cases parasitologically diagnosed were reported in recent years. The number of infected cattle was less than ten since 2016 [6]. Furthermore, more than 95% of endemic counties have achieved the criteria of transmission interruption or elimination [7].

Snail control was the predominant measure to prevent infections before application of praziquantel in P.R. China and still one of the core components of the national programme. The nationwide snail-infested areas declined from 8.2 billion m^2^ in the late 1950s to 3.6 billion m^2^ in 2020, and 201 out of 453 endemic counties are free of *Oncomelania hupensis*, the only intermediate host snail species for *S. japonicum*. Snail control contributed to achievement in schistosomiasis control in P.R. China.

Historically, more than 90% of snail-infested areas were distributed in the middle and lower reaches of Yangtze River [8]. Even today, approximately 97.4% of 3.6 billion m^2^ snail-infested areas are there [8]. *Oncomelania* snails normally infest the marshlands of the Yangtze River and major branches as well as flooding plains. Of note, *Oncomelania* snails are amphibious. The female snails lay eggs into wet soil that is washed into freshwater bodies, often during flooding events, where they hatch. Schistosomiasis transmission is hence sensitive to hydrological events. Water conservancy projects against flooding can change the hydrological conditions through fluctuation of water levels in reservoirs and reducing run-off downstream dams that impose a devastating impact on the snail populations [9–11]. Development of agricultural irrigation systems is also playing an important role in governing the distribution of *O. hupensis*, as artificial barriers were added to original water networks [12]. Other environmental modifications include replacing old irrigation ditches with new cemented canals, construction of water storages for fishing and irrigation and change of land use, among others [13].

Yangtze River valley, the major endemic area of schistosomiasis, witnessed a variety of water conservancy development [14]. In 1949–1957, embankments or levees were strengthened along the Yangtze River to deter flooding and construction of flood diversion and storage systems were undertaken [14]. Levees to a large extent blocked the spread of *Oncomelania* snails from the river and lakes to irrigation region, which facilitated the elimination of snails in the latter. In the following 20 years (1958–1977), many small reservoirs and irrigation systems abruptly rose to ensure the water supply for agriculture, which ran together with land reclamation. The snails disappeared soon after dam construction [10, 11], which showed converse impact as schistosomiasis was caused by other species [15]. The fastest elimination of snail habitats occurred in the 1960s and 1970s [16], which was consistent with the development of agriculture and water conservancy [17, 18].

In this paper, we assess the effectiveness of levees along Yangtze River and affiliated lakes by comparing the spatiotemporal dynamics of *O. hupensis* inside and outside the river.

## Methods

### Data collection

The study area covered seven provinces in the middle and lower reaches of the Yangtze River, namely Shanghai, Zhejiang, Jiangsu, Anhui, Jiangxi, Hubei and Hunan. A snail habitat was defined as a relatively independent physical environment infested by *O. hupensis*, with adjacent habitats separated by obvious barriers or with essentially different shapes. A historical or extinct habitat was that in which *Oncomelania* snails were not found in two or more consecutive years. Otherwise, the habitat was considered as existing or extant.

A habitat could be partially infested by *O. hupensis*. The term ‘snail-infested range’ was an indicator of the actual distribution of snails in a habitat. Hence, a snail-infested range was less or equal to the overall area of habitat. Since the actual distribution of snails in a habitat may change by years, the term ‘accumulated snail-infested range’ (ASR) was used to define the maximum distribution range in a habitat by overlapping annual actual distribution ranges from the time when *Oncomelania* were first observed.

The habitat survey was conducted by skilled staff in local official centres for disease control and prevention (CDCs) or institutes of schistosomiasis control (ISCs). All habitats documented by annual records were considered as the subjects in the survey, and each habitat was coded by a unique identification number (ID). The information of a habitat was extracted from annual records, for example, habitat landscape type (i.e. marshland, water network or mountainous/hilly landscape), habitat size, years for first discovery and elimination of *O. hupensis*, accumulated snail-infested range and existing snail-infested range. Data were input into a Microsoft Excel spreadsheet. Statistical analysis was performed by SPSS version 19.0 (IBM Corp.; Armonk, NY, USA).

The habitat shape was determined by global positioning system (GPS) and organised by a geographical information system (GIS). Small habitats were drawn up by tracing with hand-held GPS in WGS1984. For larger habitats, the coordinates of key boundary points were obtained by GPS and thereby habitat shapes were determined on the basis of remotely sensed images in a GIS platform. The attribute and shape data of habitat were linked by their unique ID in ArcGIS version 10.1 (ESRI, Redlands, CA, USA).

The current distribution of *Oncomelania* snails was determined by field sampling survey, which was carried out in the spring and fall of 2016 and 2017. The systematic or equal interval sampling survey was conducted in existing habitats (Fig. S1). The interval between sampling frames can be variable from 5 m to 50 m according to the habitat area or length. Environmental sampling was used in historical habitats, where the environment most likely infested by *Oncomelania* snails was investigated on the basis of the soil humidity and vegetation. The historical habitats were surveyed by systematic sampling if *Oncomelania* snails were found in environmental sampling survey. The sampling frame was fixed to 0.33 × 0.33 m according to the handbook of schistosomiasis control [19]. The average snail density was only determined by the systematic sampling result and defined as the number of living *Oncomelania* snails divided by the number of sampling frame (0.33 × 0.33 m) in the habitat. *Oncomelania* snails were collected by skilled staff with tweezers and identified according to morphological features.

The area of snail-infested range was determined by standard methods [20]. These methods were established at the early of national control programme and are still used to date. The calculation of the area can be referred to the supplementary file (Fig. S2).

### Mapping levee along Yangtze River and major branches

The middle and lower reaches of the Yangtze River is typical flooding plain. To mitigate the impact of flooding, many levees were established along the river and the major branches. The circular levees were also established in the freshwater lakes, for example, Poyang Lake and Dongting Lake, for land reclamation in 1950s–1970s. We localised the typical levees by GPS and characterised the surface features in remote sensing image. We extracted the levees on the basis of the features and thereafter divided the snail habitats into two groups, that is, inside and outside the Yangtze River. Of note, two or more levees could be established at the same river segment with different distance to water. The levee nearest to Yangtze River was used for the categorisation of snail habitats.

### Data analysis

Joinpoint regression analysis divides the study time span by connection points and further separately characterises the regression in each time period to describe the time variation trend of the research factors. In this study, the change trend of snail-infested range in different periods were analysed using Jointpoint Regression Program version 4.9.1.0, with the annual existing snail-infested range as the dependent variable and year as independent variable.

The 5-year-period moving averages of eliminated area infested by snails were calculated for habitats inside and outside Yangtze River. The corresponding geographical median centres were determined and thereby the moving routes of the centres were depicted in ArcGIS version 10.1. The moving patterns for eliminated habitats inside and outside Yangtze River were compared.

The hotspots for the snail density and population size were explored. The snail density was previously defined. Population size was estimated on the basis of the multiplication of snail density and existing area infested by snails. Fixed distance was chosen as the conceptualisation of spatial relationships and the threshold distance was determined by increment spatial autocorrelation in ArcGIS version 10.1. Hotspot analysis (Getis-Ord Gi*) of ArcGIS was used to determine the areas with statistical significance clustering of *O. hupensis* density in the Yangtze River Basin.

## Results

### Spatial distribution

A total of 216,182 snail habitats were recorded in the survey. The majority (82.04%) of habitats were located in the flooding and waterlogging areas (Fig S3). There, 7536 *O. hupensis* habitats inside the Yangtze River were identified, responsible to a total environmental area of 5071.96 million m^2^. By 2017, ASR of *O. hupensis* inside the Yangtze River was 4710.77 million m^2^. The existing snail-infested range was 3079.35 million m^2^, which implied that 34.63% ASR had been eliminated. The common habitat type inside the Yangtze River was marshland, accounting for 64.72% of habitats (Fig. S4). Environmental type of most habitats (82.13%) inside Yangtze River has not changed since the 1950s.

**Fig. 1:**
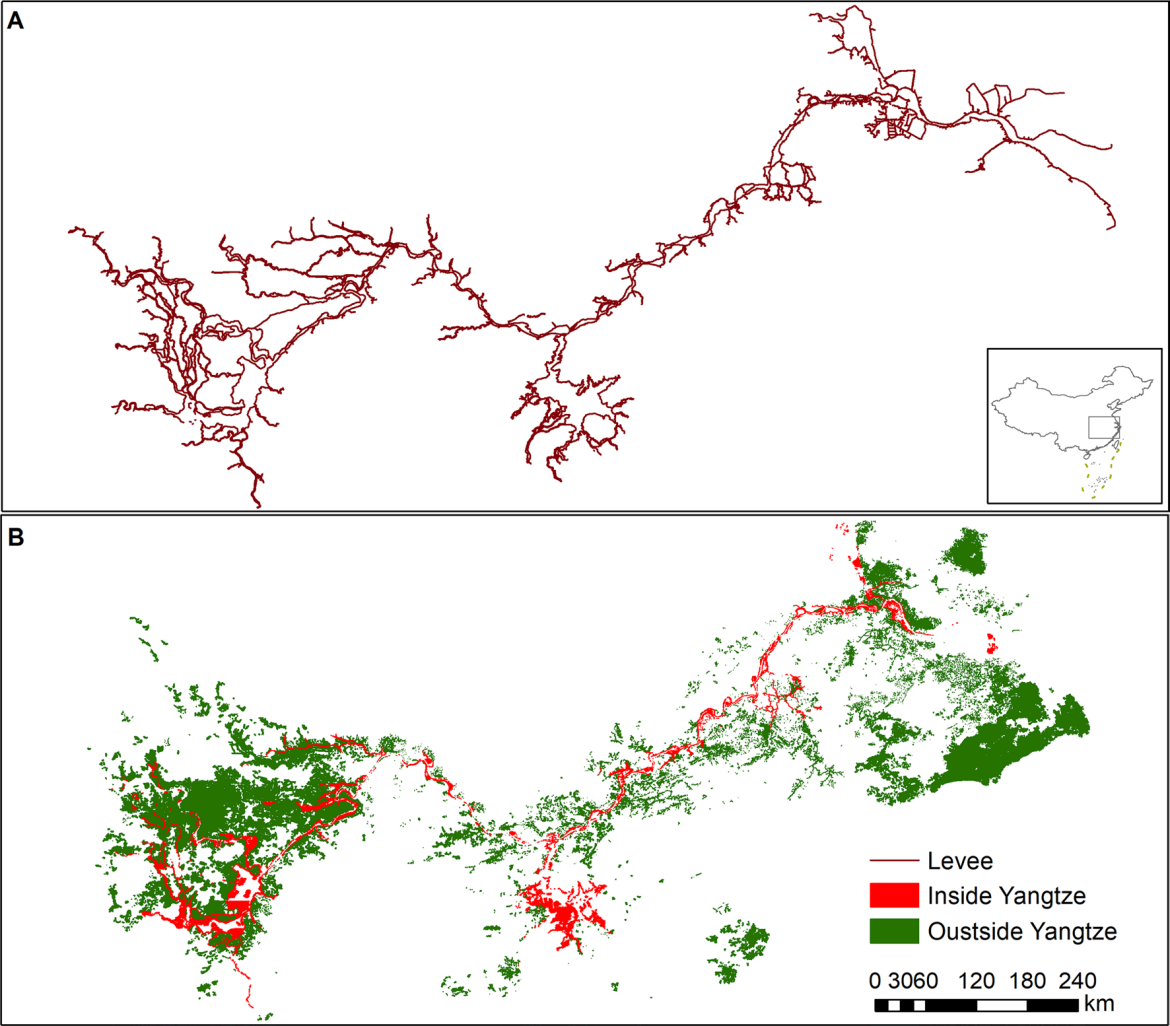
Distribution of *O. hupensis* habitats in the middle and lower reaches of Yangtze River. The flooding levees (**A**) divide the snail habitats into inside and outside groups (**B**)

There were 208,646 snail habitats outside the Yangtze River, about 27.69 times as many as inside the Yangtze River (Fig. 1). However, the total environmental area was 7681.85 million m^2^, which was about only 1.51 times of the total environment area inside the Yangtze River. By 2017, ASR of *O. hupensis* outside the Yangtze River was 7470.59 million m^2^, of which 93.83% had been eliminated (Fig. 1). The highest proportion of environmental types outside the Yangtze River was ditches, accounting for 44.66% of the total, while marshland was only 8.25%. Outside the Yangtze River, the change of habitats due to environmental modification accounted for 43.57% of the total environment, which was significantly higher than that inside the Yangtze River.

### Temporal dynamics

**Fig. 2:**
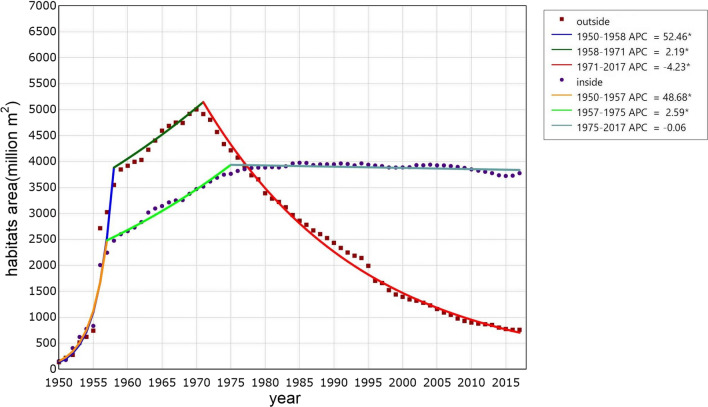
Dynamics of existing snail habitats inside and outside the Yangtze River from 1950 to 2017

The annual existing snail-infested range both inside and outside the Yangtze River significantly changed between 1950 and 2017. Three periods were identified by Joinpoint regression analysis in both groups of snail habitats (Fig. 2). In the 1950s, *O. hupensis* habitats were continuously discovered, since a nationwide survey on schistosomiasis was accomplished between 1956 and 1958. That led to the rapid increase of annual snail-infested range in this stage either inside or outside the river. The national programme of schistosomiasis control commenced after the nationwide survey, which achieved continuous discovery of snail habitats. However, the growth rate of annual existing snail-infested range decreased. The snail-infested range outside and inside the Yangtze River arrived at the corner around 1971 and 1975, respectively. After that, the annual existing snail-infested range outside the Yangtze River decreased rapidly, while that inside the Yangtze River did not change significantly. Since 1978 the snail-infested range inside exceeded that outside the Yangtze River.

**Fig. 3:**
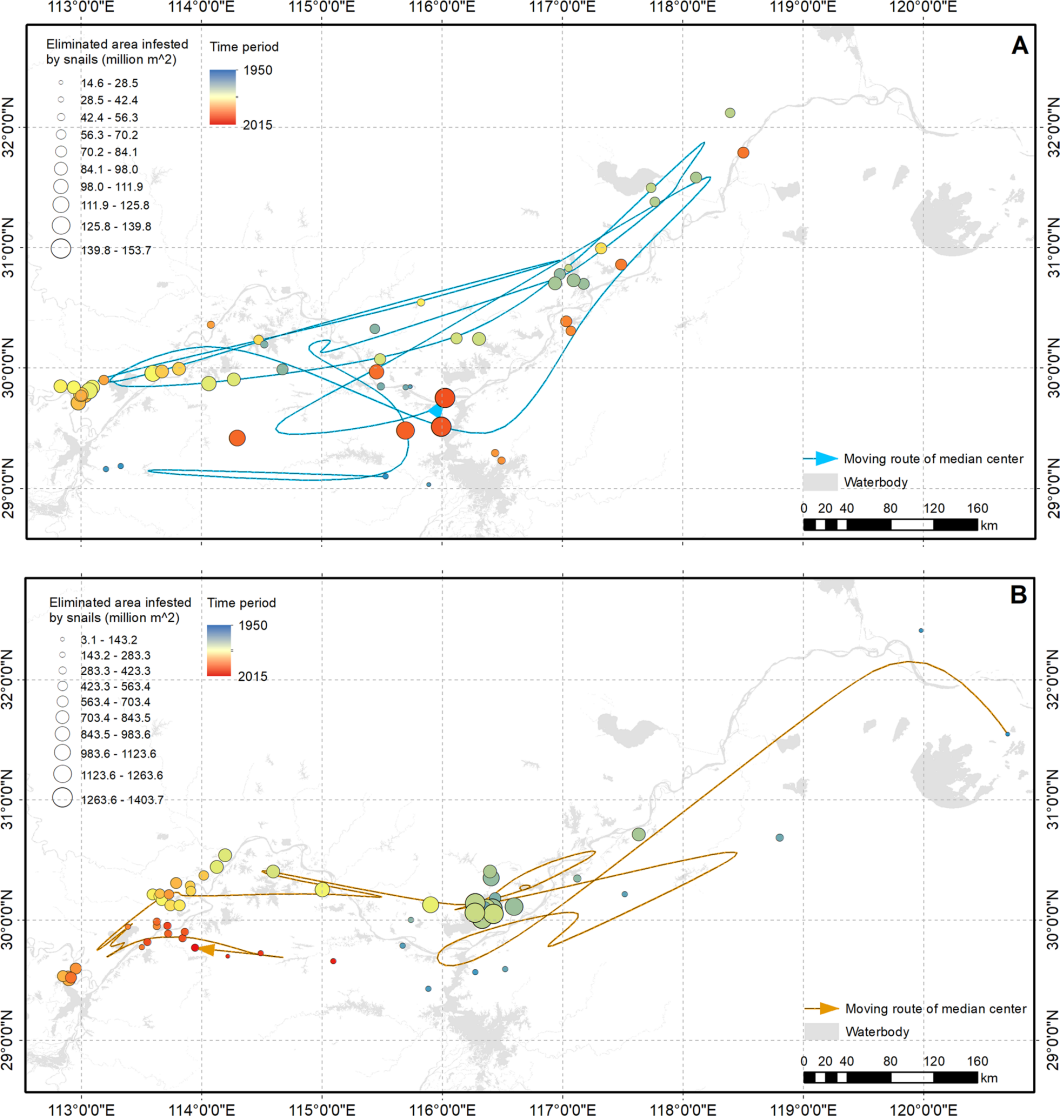
Moving route of geographical median centre of eliminated area infested by snails. **A** inside, **B** outside

The elimination pattern of snail-infested ranges showed difference inside and outside the Yangtze River (Fig. 3). The majority of eliminated ranges inside the river in the early stage were located in Poyang and Dongting lakes. The median centre for eliminated ranges then wavered between the east (lower reach) and the west (middle reach). The median centre moved to Poyang Lake in recent years, which indicated that the eliminated snail-infested ranges in the lake contributed most. The eliminated area infested by snails showed a growing trend. In contrast to snail habitats inside Yangtze River, the median centre for eliminated ranges continuously moved from the east to the west. The eliminated area infested by snails was higher in 1970s and 1980s. Although the median centre was stably located in Hubei province in recent years, the area of eliminated range was very low.

### Current hotspots

**Fig. 4:**
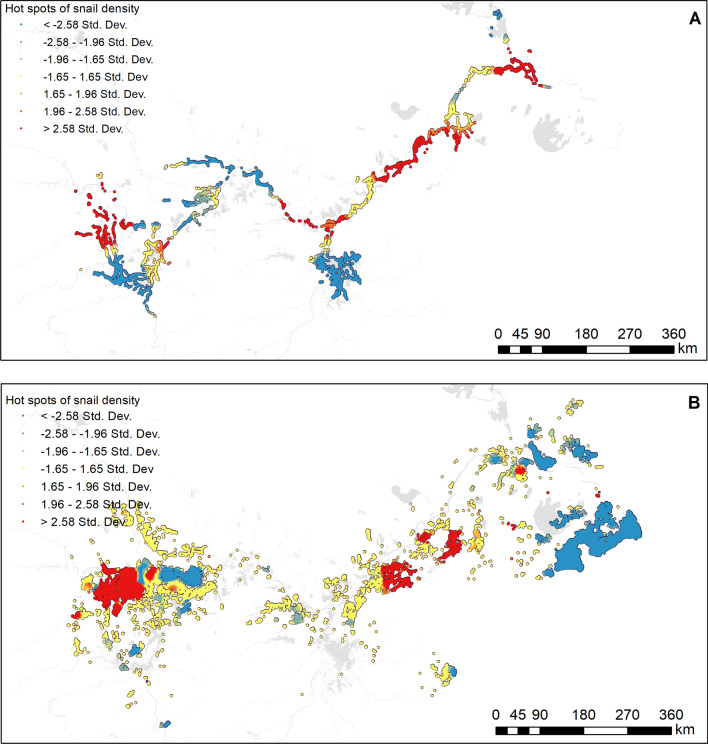
Hotspot analysis of *O. hupensis* density inside (**A**) and outside (**B**) the Yangtze River

According to systematic sampling, 3,985,105 sampling frames were set up inside Yangtze River. A total of 615,623 living *O. hupensis* were found. The average density of living *O. hupensis* was 0.154/0.1 m^2^ (Table 1). As shown in Fig. 4A, hotspots of *O. hupensis* density were scattered in the Yangtze River, while cold spots were relatively concentrated in the two biggest freshwater lakes, that is, the Poyang and Dongting.

**Table 1:** Characteristics of *O. hupensis* habitats inside and outside the Yangtze River

Indicators	Inside	Outside
Number of snail habitats	7536	208,646
Environment area (million m^2^)	5071.96	7681.85
Average area (m^2^)	673,030.78	36,817.62
Accumulated snail-infested range (million m^2^)	4710.77	7470.59
Number of existing snail habitats	4518	22,496
Existing snail-infested range (million m^2^)	3079.35	460.70
Median snail density in existing habitats	0.154	0.151

Outside the Yangtze River, 18,977,779 systematic sampling frames were screened for *O. hupensis*, and finally, 2,884,602 living *O. hupensis* were discovered. The average density of live *O. hupensis* was 0.151/0.1 m^2^, which was the same as inside the Yangtze River. As shown in Fig. 4B, hotspots of *O. hupensis* density were mainly located in Hubei and Anhui provinces, while cold spots were relatively concentrated in the delta of the Yangtze River.

## Discussion

Approximately 73% ASR has been eliminated in P.R. China since the 1950s [8], which remarkably contributed to schistosomiasis control and elimination [21]. The large-scale snail control was owed to flooding management in the last seven decades, including levee construction along the Yangtze River and major branches in the middle and lower reaches as well as the reservoir groups in the Yangtze River valley [22, 23].

The middle and lower reaches of the Yangtze River is the major flooding area in P.R. China, and also the most widely infested by *Oncomelania* snails [8]. The flood plain is highly consistent with the geographical distribution of *Oncomelania* snails, implying that the habitats of *Oncomelania* snails are closely related to flooding.

Flooding causes the connection between aquatic ecosystems in rivers and flood plains [24–26]. When flooding occurred, the connectivity of different habitats within the flood plain increased, which allowed for aquatic animals (e.g. *Oncomelania* snails) to migrate between habitats. In contrast, when the flooding receded, the connectivity between the habitats was interrupted. Levee construction along rivers is meant to prevent flooding, but inevitably affects the ecology of the flood plain. Levee causes a reduction in the frequency and intensity of flooding and separates the flood plain from the river. For aquatic animals, the decrease of hydrological connectivity limits migration and dispersal. Rapid decline of *Oncomelania* population in the flood plain of the middle and lower reaches of the Yangtze River is attributed to levee construction in the 1950s–1970s [27]. It was estimated that more than 30,000 km of levees were constructed or reinforced in the middle and lower reaches to date [27, 28].

Sluices are included in the supplementary water facility along with the development of levees. Most of the natural and artificial channels, which were connected to the Yangtze River and major branches, have a sluice at the joint [29]. Although the water can be exchanged between channels and the Yangtze River, the bank of channels is not suitable for *Oncomelania* infestation [30]. The function of a sluice is to control water levels and hence changes the original hydrology suitable for *Oncomelania* infestation.

There are 51,200 reservoirs in the Yangtze River valley, which hold the water volume of 359 billion m^3^ [28]. The reservoirs play a crucial role in fighting against floods in the middle and lower reaches. More recently, more than 40 reservoirs in the upper and middle reaches of the Yangtze River were included in the joint operation of the multi-reservoir system, which was developed and implemented in 2012 [31]. The total water volume of the reservoirs is 184 billion m^3^, including a flood control reservoir capacity of 57.4 billion m^3^ [28]. The ability of control and management of floods in the Yangtze River was further improved. Interconnectivity between the habitats of *Oncomelania* snails in the Yangtze River and flood plain was maintained, which accelerated the extinction progress of the isolated habitats in the flood plain.

The construction of levees, reservoirs and sluices indeed separated the habitats of *O. hupensis* between the Yangtze River and flood plains. Our results supported the separation, namely, the habitats inside and outside the Yangtze River showed different dynamic profiles in the last several decades. The existing snail-infested range outside the Yangtze River has been reduced by 90.84% from the peak (5031.01 million m^2^) around 1970, while that inside the Yangtze River maintained a high level of 3893.08 million m^2^ since 1975. The habitat fragmentation due to urbanisation and land reclamation accelerated the degradation of original habitats [32]. Snail control, including implementation of molluscicide and environmental modification, also contributed to the decrease in snail-infested range outside the Yangtze River [13]. On the contrary, little impact due to socio-economic development and snail control measures was observed in the habitats inside the Yangtze River and the major branches. No conclusive evidence indicated shrinkage of snail-infested range caused by the hydrological change due to plenty of reservoirs in the Yangtze River valley. However, habitat shift and snail density decrease were indeed observed inside the Yangtze River [33]. Large-sized habitats and good connectivity led to stable snail habitats inside the river and branches.

Some recommendations were made according to our findings. First, a sluice system was recommended at the inlets of branches of the Yangtze River. Although levees have been built along the banks of major rivers and lakes, some smaller branches are open to the Yangtze River and lakes without any cut-off facility. The snail density and habitat area are often high in such branches according to the present hotspot analysis. In this case, a sluice system should be considered at the joint.

Second, we suggested intensive surveillance in some regions. According to moving route of eliminated area infested by snails, the major reduction of area infested by snails moved to middle stream of Yangtze River and the annual decrease of area infested by snail is becoming smaller by year, which means that a hard nut was encountered. The habitats outside the Yangtze River are mainly distributed in irrigation system across the middle and down streams of the river. The majority of habitats in the downstream of Yangtze River were eliminated before the 1970s. In contrast, only a small proportion of habitats outside the Yangtze River in the middle stream was eliminated [34], with worse waterlogging in the plains of the middle stream due to elevated river bed caused by sediment deposition being one of the major reasons [35]. Intensive surveillance on source of infections should be enforced in such areas.

Third, monitoring and evaluation should be implemented in Poyang and Dongting lakes. According to hotspot analysis, the major code spots were located in the two lakes. However, the snail density of the marshland in the lakes was high before the Three Gorges Dam operation [36]. The previous studies showed that new habitats had emerged since the water level in the lakes became lower after the Three Gorges Dam operation [33]. The belts of high density might be re-established in the future. Therefore, the spatial distribution pattern of *Oncomelania* snails should be monitored and evaluated to make effective strategies for snail control.

Finally, the relation between geographic distribution of *S. japonicum* and *O. hupensis* shall be analysed further. Although snails are not the only factor that influences the distribution of *Schistosoma*, it would be interesting to compare the geographic range of both the snail host and the parasite species.

## Conclusions

The present study showed the difference pattern inside and outside the Yangtze River in three aspects, that is, spatial distribution, temporal dynamics and current hotspots. Our results indicated that the levees had a considerable negative impact on the distribution of *O. hupensis* outside the Yangtze River, while they had limited influence on the snails inside the river. The findings from hotspot analysis suggested a sluice system at the inlet of branch. The major distribution of *O. hupensis* located in Hubei might be caused by severe waterlogging. Since human and animals are ready to access the *Oncomelania* snails in the areas, intensive surveillance should be implemented there. The two biggest freshwater lakes, the historical major endemic regions, were identified as cold spots. The long-term impact of the Three Gorges Dam on the distribution of *O. hupensis* in the lakes should be monitored and evaluated.

### Supplementary Information

Supplementary material 1: Figure S1. Survey methods on the distribution of *Oncomelania*.

Supplementary material 2: Figure S2. Calculation methods of the distribution area of *Oncomelania*.

Supplementary material 3: Figure S3. Distribution of *Oncomelania hupensis* in flooded and waterlogged areas in the middle and lower reaches of Yangtze River.

Supplementary material 4: Figure S4. The three landscape types of *Oncomelania hupensis* habitats in the middle and lower reaches of Yangtze River.

## Data Availability

The data from the national survey on *Oncomelania* snail are deposited at National Institute of Parasitic Diseases, China CDC.
