# Supplementary figures and images for: Assessment of the influence of levees along Yangtze River on Oncomelania hupensis, the intermediate host of Schistosoma japonicum
Source: Parasit Vectors. 2024 Jul 7;17:291. doi: 10.1186/s13071-024-06318-1 (PMC11229218; doi:10.1186/s13071-024-06318-1)

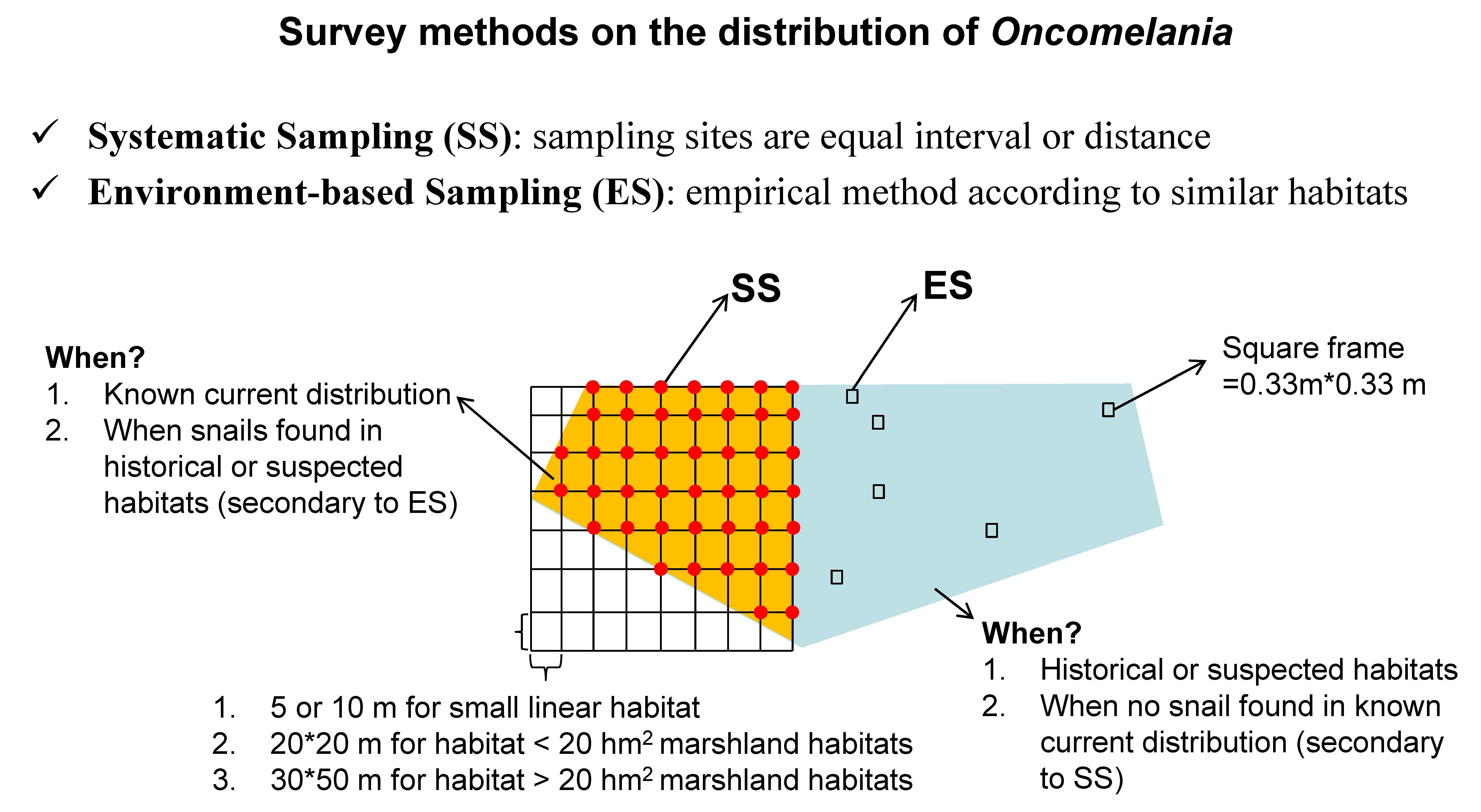

Supplement: Supplementary file 1 — Supplementary material 1: Figure S1. Survey methods on the distribution of Oncomelania. [file 13071_2024_6318_MOESM1_ESM.jpg]

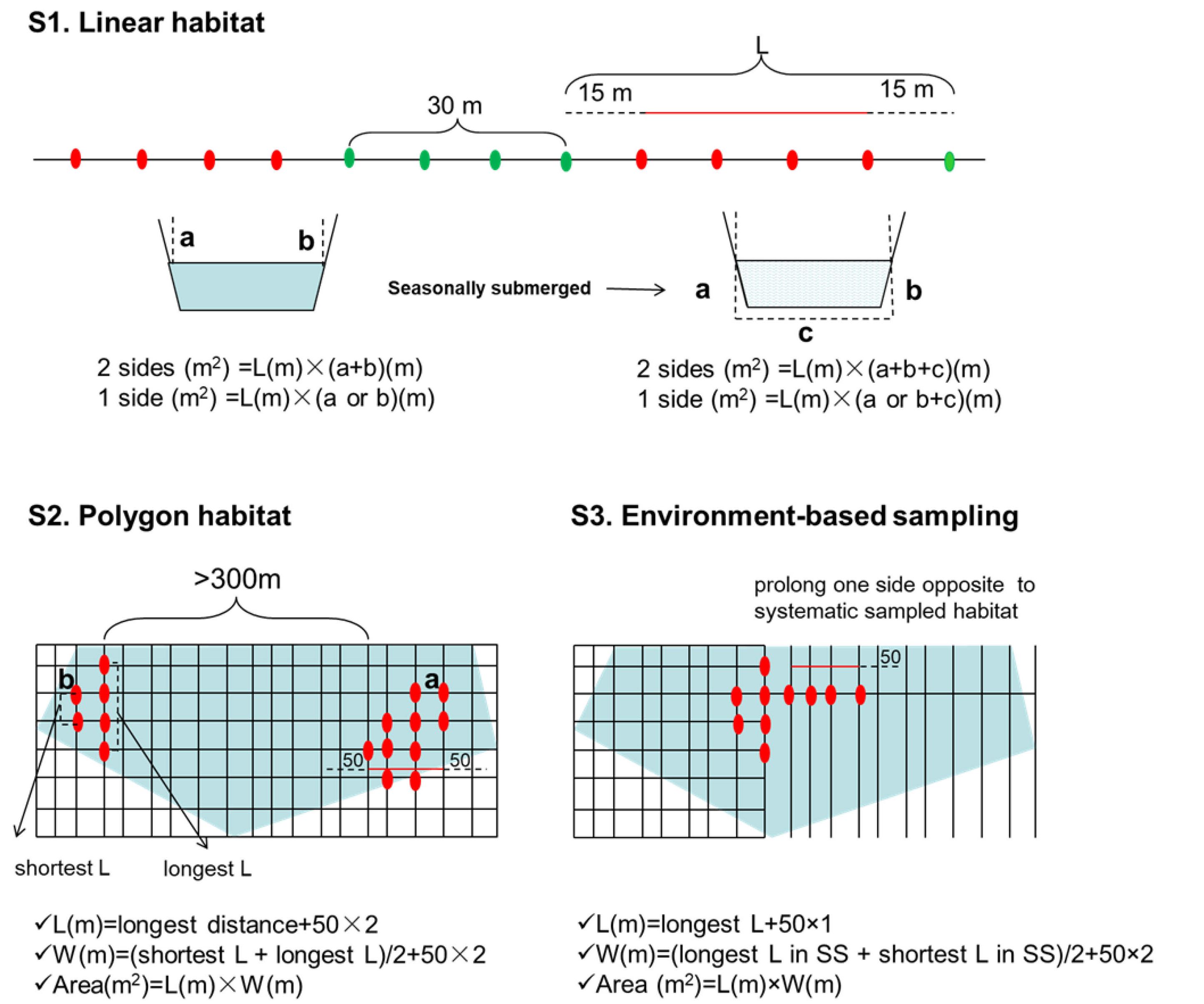

Supplement: Supplementary file 2 — Supplementary material 2: Figure S2. Calculation methods of the distribution area of Oncomelania. [file 13071_2024_6318_MOESM2_ESM.jpg]

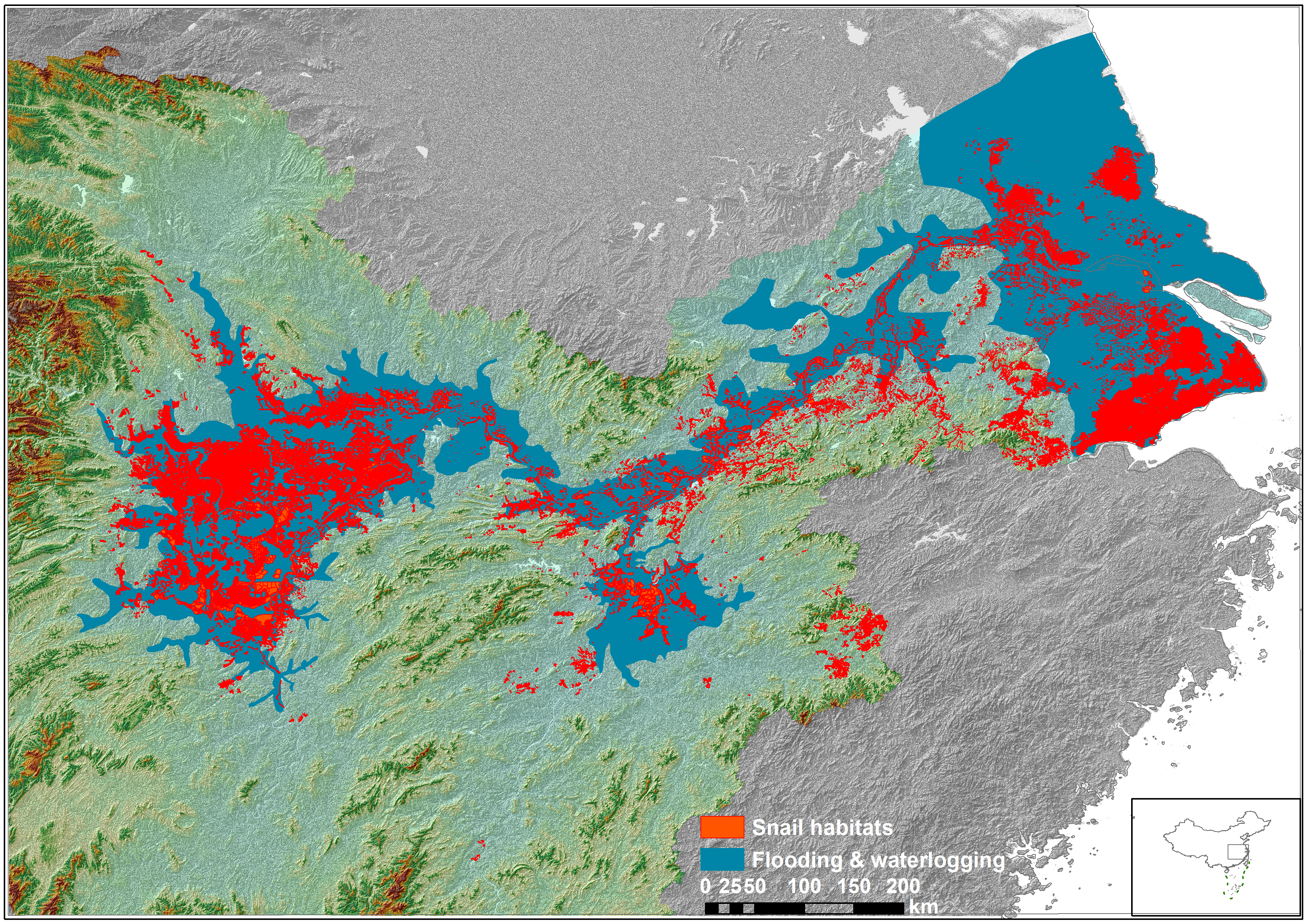

Supplement: Supplementary file 3 — Supplementary material 3: Figure S3. Distribution of Oncomelania hupensis in flooded and waterlogged areas in the middle and lower reaches of Yangtze River. [file 13071_2024_6318_MOESM3_ESM.tif]

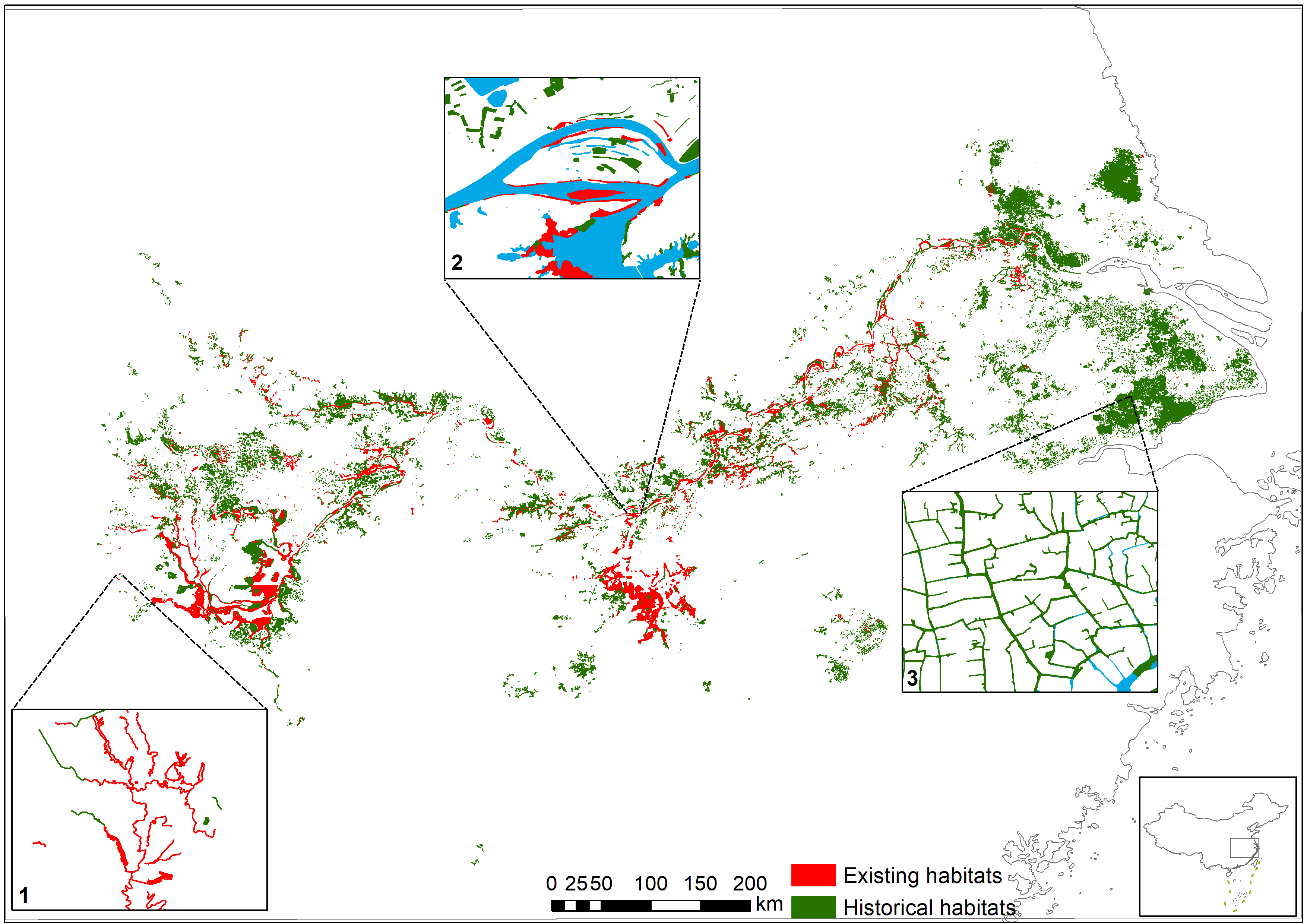

Supplement: Supplementary file 4 — Supplementary material 4: Figure S4. The three landscape types of Oncomelania hupensis habitats in the middle and lower reaches of Yangtze River. [file 13071_2024_6318_MOESM4_ESM.tif]
